# Induction of cytokines and growth factors by rapamycin in the microenvironment of brain metastases of lung cancer

**DOI:** 10.3892/ol.2013.1135

**Published:** 2013-01-15

**Authors:** SE HOON KIM, JUNG EUN LEE, SEUNG-HO YANG, SANG WON LEE

**Affiliations:** 1 Department of Pathology, Yonsei University College of Medicine, Seodaemun-gu, Seoul;; 2 Department of Neurosurgery, St. Vincent’s Hospital, The Catholic University of Korea, Suwon, Kunggido 442-723, Republic of Korea

**Keywords:** brain metastasis, non-small cell lung cancer, rapamycin, astrocytes, microenvironment

## Abstract

The association between rapamycin and astrocytes in a tumor-bearing mouse model with brain metastases of non-small cell lung cancer (NSCLC) was investigated. For *in vitro* experiments, NCI-H358, a human lung adenocarcinoma cell line, was co-cultured with immortalized astrocytes, and treated with rapamycin, an mTOR inhibitor. We evaluated the expression of interleukin-1 (IL-1), interleukin-3 (IL-3), interleukin-6 (IL-6), tumor necrosis factor-α (TNF-α), transforming growth factor-β (TGF-β), insulin-like growth factor-1 (IGF-1), platelet-derived growth factor (PDGF), chemoattractant protein-1 (MCP-1) and macrophage inflammatory protein-1 (MIP-1) in tumor cells *in vivo*. Rapamycin is cytotoxic *in vitro*; however, co-culturing tumor cells and astrocytes induced tumor cell survival. IL-1, IL-3, IL-6, TNF-α, TGF-β, PDGF, MCP-1 and MIP-1 expression were higher in rapamycin-treated mice compared to the control group, however, IGF-1 expression was lower. Notably, treatment with rapamycin before inoculating tumor cells affected cytokine expression in the tumor microenvironment. We suggest that growth factors and cytokines in the tumor microenvironment play a role in the survival of cancer cells in brain metastases.

## Introduction

Lung cancer is the leading cause of cancer-related mortality in the United States and Europe, and 80% of cases are diagnosed as non-small cell lung cancer (NSCLC). Although NSCLC frequently develops brain metastasis, effective treatment and prevention for brain metastasis remain unavailable (1,2).

Metastases form when specialized tumor cells (‘seeds’) find a suitable environment (‘soil’) to arrest, invade and grow (3). Metastasis patterns to different organs are dependent on the tumor cell phenotype and interactions between the tumor cell and the environment, such as cellular components, cytokines and organ-derived growth factors (4).

In the central nervous system, glial cells, which have traditionally been viewed as providing structural support for neurons, also play an important role in maintaining homeostasis. Astrocytes support immune defense in the brain and protect neuronal cells from waste products and hypoxic damage. Astrocytes also produce a wide variety of cytokines, including interleukin-1 (IL-1), interleukin-3 (IL-3), interleukin-6 (IL-6), tumor necrosis factor-α (TNF-α), transforming growth factor-β (TGF-β), insulin-like growth factor-1 (IGF-1) and platelet-derived growth factor (PDGF). Cytokines released by activated microglia include IL-1, IL-6, TNF-α, monocyte chemoattractant protein-1 (MCP-1) and macrophage inflammatory protein-1 (MIP-1). MCP-1 recruits NK cells and a subpopulation of T-lymphocytes. MIP-1 attracts activated/memory T-cells, monocytes/macrophages, and immature dendritic cells (5–7).

It has recently been postulated that maintaining E-cadherin expression could potentially block increases in motility and invasion during epithelial-to-mesenchymal transitions (EMT). It has further been suggested that the brain microenvironment could be affected by pre-treatment with peroxisome proliferator-activated receptor γ (PPARγ)-activating drugs, even before brain metastasis (8).

Rapamycin, a lipophilic macrolide antibiotic, was originally identified as a fungicide and immunosuppressant. Studies have revealed, however, that rapamycin can potently arrest the growth of cells derived from a broad spectrum of cancers. Rapamycin has been shown to specifically inhibit mammalian target of rapamycin (mTOR), which is a key player in tumor development and progression (9). Rapamycin can impede tumor metastasis by suppressing tumor angiogenesis and lymphangiogenesis (10,11).

A dynamic interaction likely exists between cancer cells and the host microenvironment to support cancerous growth and spread. The aim of this study was to identify interactions between astrocytes and rapamycin in brain metastases of NSCLC.

## Materials and methods

### Apoptosis assay

NCI-H358, a human lung adenocarcinoma cell line, was obtained from the American Tissue Culture Collection (Manassas, VA, USA). NCI-H358 tumor cells (1×10^4^ cells/well) were plated in 96-well flat bottomed tissue culture plates and incubated at 37°C in a 5% CO2/95% air atmosphere. Rapamycin (10 or 100 *μ*g/ml) was then added to tumor cells cultured alone or co-cultured with astrocytes. Following 24, 48 or 72 h rapamycin treatment, 10 *μ*l MTT (3-(4,5-Dimethylthiazol-2-yl)-2,5-diphenyltetrazoliumbromide) stock solution (EZ Cytox, Daeil Lab Service Co., Seoul, Korea) was added to each well, and the plates were incubated for 4 h. Plates were agitated on a plate shaker for 3 sec, and the absorbance at 540 nm was determined using a scanning multi-well spectrophotometer (VERSA max, Sunnyvale, CA, USA).

### Co-culture of astrocytes and NCI-H358, and rapamycin administration

Immortalized astrocytes were plated on sterile 0.4 μm cell culture inserts (Becton-Dickinson Labware, Franklin Lakes, NJ, USA) at a density of 4×10^5^ cells in 1 ml Dulbecco’s modified Eagle’s medium (DMEM) containing 10% fetal bovine serum (FBS). The inserts were gently transferred to empty 6-well plates and placed in at 37°C. NCI-H358 lung adenocarcinoma cells were plated in 6-well plates at a density of 5×10^5^ cells/well in 2 ml of RPMI-1640 containing 10% FBS. After incubating overnight, the medium of both cultures was removed and replaced with serum-free medium. Inserts containing astrocytes were transferred to 6-well plates containing tumor cells and co-cultured for 24, 48 and 72 h. Control samples consisted of cell-free inserts placed in 6-well plates containing tumor cells. At each time point the inserts were discarded and the tumor cells were washed with ice-cold PBS and lysed with buffer. NCI-H358 cells alone and co-cultured with astrocytes were treated with rapamycin (100 g/ml) 24 h later.

This study was approved by the Ethics Committee of St. Vincent’s Hospital, The Catholic University of Korea, Suwon, Korea.

### Inoculation of intracranial cancer cells and experimental design

The nude mice were anesthetized with an intraperitoneal (i.p.) injection of 12 mg/kg xylazine (Rompun; Cutter Laboratories, Shawnee, KS, USA) and 30 mg/kg ketamine (Ketalar; Parke-Davis & Co., Morris Plains, NJ, USA). The mice were then stereotaxically inoculated with 1×10^6^ NCI-H358 cells into the right frontal lobe (2 mm lateral and 1 mm anterior to the bregma, at a depth of 2.5 mm from the skull) using a sterile Hamilton syringe fitted with a 26-gauge needle (Hamilton Co., Reno, NV, USA) and a microinfusion pump (Harvard Apparatus, Holliston, MA, USA). Intracranial tumors were confirmed by cranial magnetic resonance imaging (MRI). All MRI experiments were performed on a 4.7 T animal MRI scanner (BioSpec 47/40, Bruker, Germany) with a quadrature volume coil (diameter, 25 mm) at the Korea Basic Science Institute in Ochang, Korea.

**Figure 1:**
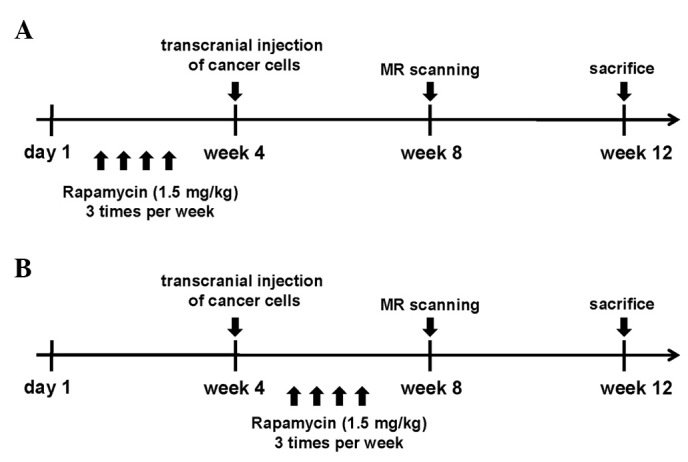
Therapeutic schedule.

The experimental design is shown in Fig. 1. Both experimental groups contained five mice initially. In the first group, mice were treated with rapamycin (1.5 mg/kg) via i.p. injection 3 times a week for 4 weeks before intracranial inoculation with NCI-H358 cells. In the second treatment group, mice were treated with rapamycin (1.5 mg/kg) via i.p. injection 3 times a week for 4 weeks after intracranial inoculation. Control and treated mice were euthanized 12 weeks after the intracranial inoculation, and tumor specimens were obtained for RT-PCR.

### Reverse transcription-polymerase chain reaction (RT-PCR)

Total RNA from all specimens was extracted using a commercial kit (RNeasy Mini kit, Qiagen, Hilden, Germany). One microgram of total RNA was reverse transcribed using the RT-premix (M-Biotech, Seoul, Korea). RT-PCR was performed on cDNA samples using a DNA Thermal Cycler (Bio-Rad, Hercules, CA, USA) with Go Taq Green Master mix (Promega, Madison, WI, USA), RNase-free water and primers. The primer sequences are summarized in Table I. RT-PCR products were separated on a 1.5% agarose gel containing ethidium bromide and visualized with UV light.

### Statistical analysis

All data are shown as the means ± SEM. Comparisons between groups were made using unpaired Student’s t-tests, and among multiple groups by ANOVA. P<0.05 was considered to indicate a statistically significant result.

## Results

### Astrocytes and rapamycin induce apoptosis of NCI-H358 cells

**Figure 2:**
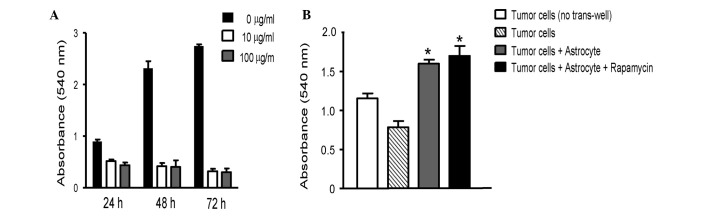
(A) The effect of rapamycin on NCI-H358 cell growth. NCI-H358 cells were treated with 10 or 100 *μ*g/ml rapamycin for 24, 48 or 72 h. Cell growth was determined by methyl thiazolyl tetrazolium assay (MTT). Rapamycin inhibited survival of NCI-H358 lung cancer cells in a dose- and time-dependent manner. 0 *μ*g/ml of rapamycin represents the control group. (B) Co-culturing NCI-H358 cells and immortalized astrocytes induced tumor cell survival, even with rapamycin treatment. Data are presented as mean ± SEM (n=6 wells per measurement). ^*^P<0.05 vs. tumor cells only.

Rapamycin inhibits survival of NCI-H358 lung cancer cells in a dose- and time-dependent manner. To determine whether astrocytes affect tumor cell survival, astrocytes were co-cultured with NCI-H358 lung cancer cells. Co-culture with astrocytes induced NCI-H358 cell survival, compared with NCI-H358 cells alone. Moreover, rapamycin (100 *μ*g/ml) enhanced the survival of lung cancer cells in co-culture (Fig. 2).

### Modulation of cytokines and growth factors by rapamycin in brain metastasis model

**Figure 3:**
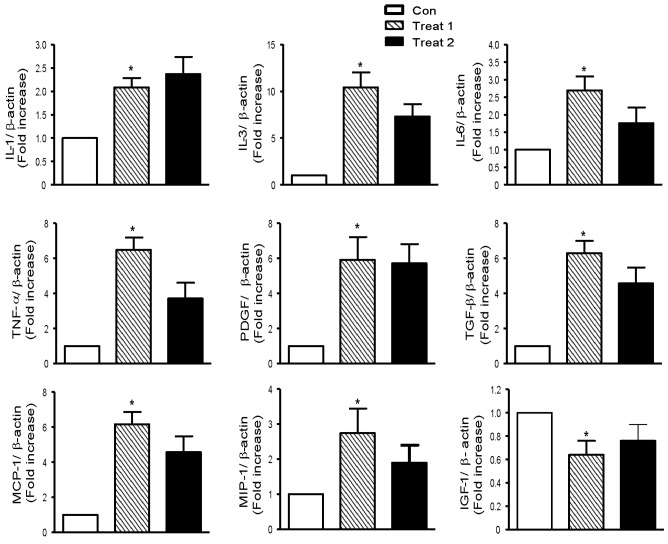
Reverse transcription-polymerase chain reaction (RT-PCR) results. Interleukin-1 (IL-1), IL-3, IL-6, tumor necrosis factor-α (TNF-α), transforming growth factor-β (TGF-β), platelet-derived growth factor (PDGF), chemoattractant protein-1 (MCP-1) and macrophage inflammatory protein-1 (MIP-1) expression increased following rapamycin administration. Expression was higher in mice treated with rapamycin before inoculation than control mice. Conversely, IGF-1 expression decreased. Data are presented as mean ± SEM (n=4 each). ^*^P<0.05 vs. control.

**Figure 4:**
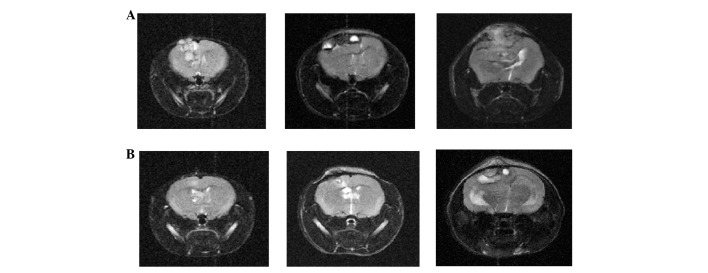
Serial magnetic resonance imaging (MRI). (A) A tumor was noted in the cortex of the right hemisphere 4 weeks after intracranial inoculation of 1×10^6^ NCI-H358 cells (left). The tumor size progressively increased and compressed the brain parenchyma at 8 weeks (middle) and 12 weeks (right) after the inoculation of control mice. (B) In mice treated with rapamycin before inoculation, the tumor formed and thrived relatively slowly. Left, 4 weeks; middle, 8 weeks; right, 12 weeks.

Activated astrocytes produce a wide variety of cytokines, including IL-1, IL-3, IL-6, TNF-α, TGF-β, IGF-1 and PDGF. Cytokines released by activated microglia include IL-1, IL-6, TNF-α, MCP-1, and MIP-1. IL-1, IL-3, IL-6, TNF-α, TGF-β, PDGF, MCP-1 and MIP-1 expression was higher in rapamycin-treated mice compared with controls (Fig. 3). IGF-1 expression, however, was lower in rapamycin-treated mice than control mice. Rapamycin treatment before tumor cell inoculation affected the later cytokine expression in the tumor. The tumor progressively increased in size, and compressed the brain parenchyma at 4, 8 and 12 weeks after inoculation in control mice. In mice treated with rapamycin before inoculation, the tumor formed and thrived slowly (Fig. 4).

## Discussion

Rapamycin caused apoptosis of tumor cells alone. However, co-cultures of astrocytes with NCI-H358 lung cancer cells prevented apoptosis of tumor cells even after rapamycin treatment. In the experimental mouse model, IL-1, IL-3, IL-6, TNF-α, TGF-β, PDGF, MCP-1 and MIP-1 expression in the inoculated tumors increased in rapmycin-treated mice. IGF-1 expression, however, decreased.

Co-culturing breast or lung cancer cells with astrocytes led to upregulated survival genes, including GSTA5, BCL2L1 and TWIST1, in tumor cells (12). Brain metastases are surrounded and infiltrated by activated astrocytes and are highly resistant to chemotherapy. Astrocytes in co-culture were activated by tumor cell-oriented factors, including macrophage migration inhibitory factor (MIF), IL-8 and plasminogen activator inhibitor-1 (PAI-1). Interactions between metastatic tumor cells and activated astrocytes are important in creating a favorable microenvironment for the tumor cells in the brain (13).

The expression of cytokines and growth factors in the tumor-bearing mouse model was measured to investigate the role of rapamycin in the microenvironment of brain metastases.

IL-1 is a macrophage-derived proinflammtory ‘alarm’ cytokine that mediates inflammation. Balanced IL-1 levels have been associated with inducing antitumor immunity (14). IL-3 gene expression within tumors leads to host-cell infiltration, particularly by macrophages, slower tumor growth and enhanced immunogenicity (15). IL-6 has been shown to inhibit growth and enhance motility in breast cancer cell lines (16). TNF-α has an antitumor effect in murine tumors and human tumor xenografts *in vivo* and appears to be cytotoxic to many human tumor cell lines *in vitro*(17). TGF-β is an important suppressor of primary tumorigenesis. The role of TGF-β in apoptosis and cell cycle arrest depends on the microenvironment (18). PDGF induces important cellular processes including chemotaxis, survival, apoptosis and transformation *in vitro*(19). MCP-1, also known as chemokine ligand 2 (CCL2), is a pro-inflammatory chemokine that recruits and activates monocytes during the inflammatory response. MCP-1 is a pivotal regulator of tumor growth, progression and metastasis (20,21). MCP-1 and MIP-1 have been reported to regulate immunity to melanoma by promoting lymphocyte infiltration into tumors and subsequent cytokine production (22,23). MCP-1 or MIP-1 loss significantly promotes primary tumor growth and lung metastasis by inhibiting IL-6, TNF-α and TGF-β expression. In this study, IGF-1 levels decreased in rapamycin-treated mice. IGF-1 is critical to activate and sustain an inflammatory response in the liver, which is needed for hepatic metastasis, not only through direct, paracrine effects on tumor cell growth, but also through indirect effects involving the tumor microenvironment (24). IGF-1 receptor expression in neuroblastoma cells has been reported to increase tumor cell interaction with the bone microenvironment, resulting in greater metastasis formation (25).

Rapamycin is highly lipophilic and thus penetrates the blood brain barrier (BBB) (26). Combined treatment with rapamycin and brain penetrant MEK inhibitor significantly reduces brain metastasis by prohibiting perivascular invasion of tumor cells and tumor angiogenesis in triple-negative breast cancer models (27). Rapamycin effectively inhibits cancer metastasis in various preclinical models (28–31).

The results demonstrated that several cytokines and growth factors, except for IGF-1, were increased in mice treated with rapamycin before inoculation, compared with control mice. Brain MRIs of tumor-bearing mice showed that tumor growth was slower in pre-treated mice than in control mice. These results suggest that rapamycin administration influences the brain microenvironment before brain metastases develop. Accumulating evidence indicates that metastatic progression is regulated, in large part, by interactions between tumor cells and non-tumor cells in the brain microenvironment (32,33). Astrocytes could contribute to the microenvironment associated with metastatic cell growth, and they are a source of cytokines and growth factors, which may modulate metastatic-cell growth. The results suggest that modulating the brain microenvironment could be worthy of further research as a new target to prevent brain metastasis of NSCLC.

## Figures and Tables

**Table I:** Primer pairs for reverse transcription-polymerase chain reaction (RT-PCR).

mRNA	Direction	Sequence	Length (bp)
IL-1	Forward	GAGAGCCGGGTGACAGTATC	
	Reverse	ACTTCTGCCTGACGAGCTTC	202
IL-3	Forward	TGCCTACATCTGCGAATGAC	
	Reverse	TTAGGTGCTCTGCCTGCTG	203
IL-6	Forward	ACTTCCATCCAGTTGCCTTC	
	Reverse	CAGAATTGCCATTGCACAAC	201
TNF-α	Forward	CCCATGGATGTCCCATTTAG	
	Reverse	CCAGGATTCTGTGGCAATC	193
ATGF-β	Forward	GTGGTTTCTGTGACCCTTGG	
	Reverse	CCAGTCACACAGGCAACAAG	201
IGF-1	Forward	CTCTTCTACCTGGCGCTCTG	
	Reverse	GCAACACTCATCCACAATGC	195
PDGF-A	Forward	CCCCTGCCCATTCGGAGGAAGAG	
	Reverse	TTGGCCACCTTGACGCTGCGGTG	203
MCP-1	Forward	CTCACCTGCTGCTACTCATTC	
	Reverse	GCTTGAGGTGGTTGTGGAAAA	650
MIP-1	Forward	TCAGCACCATGAAGGTCTCCAC	
	Reverse	CTCAGGCATTTAGTTCCAGCTC	450

## References

[1] Jemal A, Siegal R, Ward E, Hao Y, Xu J, Murray T, Thun MJ (2008). Cancer statistics, 2008. CA Cancer J Clin.

[2] Robnett TJ, Machtay M, Stevenson JP, Algazy KM, Hahn SM (2001). Factors affecting the risk of brain metastases after definitive chemoradiation for locally advanced non-small-cell lung carcinoma. J Clin Oncol.

[3] Rusciano D, Burger MM (1992). Why do cancer cells metastasize into particular organs?. Bioessays.

[4] Nicolson GL (1993). Cancer progression and growth: relationship of paracrine and autocrine growth mechanisms to organ preference of metastasis. Exp Cell Res.

[5] Sierra A, Price JE, García-Ramirez M, Méndez O, López L, Fabra A (1997). Astrocyte-derived cytokines contribute to the metastatic brain specificity of breast cancer cells. Lab Invest.

[6] Yang I, Han SJ, Kaur G, Crane C, Parsa AT (2010). The role of microglia in central nervous system immunity and glioma immunology. J Clin Neurosci.

[7] Langley RR, Fan D, Guo L, Zhang C, Lin Q, Brantley EC, McCarty JH, Fidler IJ (2009). Generation of an immortalized astrocyte cell line from H-2Kb-tsA58 mice to study the role of astrocytes in brain metastasis. Int J Oncol.

[8] Yoo JY, Yang SH, Lee JE, Cho DG, Kim HK, Kim SH, Kim IS, Hong JT, Sung JH, Son BC, Lee SW (2012). E-cadherin as a predictive marker of brain metastasis in non-small-cell lung cancer, and its regulation by pioglitazone in a preclinical model. J Neurooncol.

[9] Bjornsti MA, Houghton PJ (2004). The TOR pathway: a target for cancer therapy. Nat Rev Cancer.

[10] Guba M, von Breitenbuch P, Steinbauer M, Koehl G, Flegel S, Hornung M, Bruns CJ, Zuelke C, Farkas S, Anthuber M, Jauch KW, Geissler EK (2002). Rapamycin inhibits primary and metastatic tumor growth by antiangiogenesis: involvement of vascular endothelial growth factor. Nat Med.

[11] Kobayashi S, Kishimoto T, Kamata S, Otsuka M, Miyazaki M, Ishikura H (2007). Rapamycin, a specific inhibitor of the mammalian target of rapamycin, suppresses lymphangiogenesis and lymphatic metastasis. Cancer Sci.

[12] Kim SJ, Kim JS, Park ES, Lee JS, Lin Q, Langley RR, Maya M, He J, Kim SW, Weihua Z, Balasubramanian K, Fan D, Mills GB, Hung MC, Fidler IJ (2011). Astrocytes upregulate survival genes in tumor cells and induce protection from chemotherapy. Neoplasia.

[13] Seike T, Fujita K, Yamakawa Y, Kido MA, Takiguchi S, Teramoto N, Iguchi H, Noda M (2011). Interaction between lung cancer cells and astrocytes via specific inflammatory cytokines in the microenvironment of brain metastasis. Clin Exp Metastasis.

[14] Carmi Y, Rinott G, Dotan S, Elkabets M, Rider P, Voronov E, Apte RN (2011). Microenvironment-derived IL-1 and IL-17 interact in the control of lung metastasis. J Immunol.

[15] Wu YZ, Hong JH, Huang HH, Dougherty GJ, McBride WH, Chiang CS (2000). Mechanisms mediating the effects of IL-3 gene expression on tumor growth. J Leukoc Biol.

[16] Jiang XP, Yang DC, Elliott RL, Head JF (2011). Down-regulation of expression of interleukin-6 and its receptor results in growth inhibition of MCF-7 breast cancer cells. Anticancer Res.

[17] Suarez Pestana E, Björklund G, Larsson R, Nygren P, Nilsson K, Bergh J (1996). Effects of interferons and tumour necrosis factor-alpha on human lung cancer cell lines and the development of an interferon-resistant lung cancer cell line. Acta Oncol.

[18] Tse JC, Kalluri R (2007). Mechanisms of metastasis: epithelialto-mesenchymal transition and contribution of tumor microenvironment. J Cell Biochem.

[19] Yu J, Ustach C, Kim HR (2003). Platelet-derived growth factor signaling and human cancer. J Biochem Mol Biol.

[20] Raffaghello L, Cocco C, Corrias MV, Airoldi I, Pistoia V (2009). Chemokines in neuroectodermal tumour progression and metastasis. Semin Cancer Biol.

[21] Zhang J, Patel L, Pienta KJ (2010). CC chemokine ligand 2 (CCL2) promotes prostate cancer tumorigenesis and metastasis. Cytokine Growth Factor Rev.

[22] Nakasone Y, Fujimoto M, Matsushita T, Hamaguchi Y, Huu DL, Yanaba M, Sato S, Takehara K, Hasegawa M (2012). Host-derived MCP-1 and MIP-1α regulate protective anti-tumor immunity to localized and metastatic B16 melanoma. Am J Pathol.

[23] van Deventer HW, Serody JS, McKinnon KP, Clements C, Brickey WJ, Ting JP (2002). Transfection of macrophage inflammatory protein 1 alpha into B16 F10 melanoma cells inhibits growth of pulmonary metastases but not subcutaneous tumors. J Immunol.

[24] Wu Y, Brodt P, Sun H, Mejia W, Novosyadlyy R, Nunez N, Chen X, Mendoza A, Hong SH, Khanna C, Yakar S (2010). Insulin-like growth factor-I regulates the liver microenvironment in obese mice and promotes liver metastasis. Cancer Res.

[25] Wu Y, Yakar S, Zhao L, Hennighausen L, LeRoith D (2002). Circulating insulin-like growth factor-I levels regulate colon cancer growth and metastasis. Cancer Res.

[26] Sehgal SN, Baker H, Vézina C (1975). Rapamycin (AY-22,989), a new antifungal antibiotic. II Fermentation, isolation and characterization. J Antibiot.

[27] Zhao H, Cui K, Nie F, Wang L, Brandl MB, Jin G, Li F, Mao Y, Xue Z, Rodriguez A, Chang J, Wong ST (2012). The effect of mTOR inhibition alone or combined with MEK inhibitors on brain metastasis: an *in vivo* analysis in triple-negative breast cancer models. Breast Cancer Res Treat.

[28] Luan FL, Ding R, Sharma VK, Chon WJ, Lagman M, Suthanthiran M (2003). Rapamycin is an effective inhibitor of human renal cancer metastasis. Kidney Int.

[29] Yang Z, Lei Z, Li B, Zhou Y, Zhang GM, Feng ZH, Zhang B, Shen GX, Huang B (2010). Rapamycin inhibits lung metastasis of B16 melanoma cells through down-regulating alphav integrin expression and up-regulating apoptosis signaling. Cancer Sci.

[30] Patel V, Marsh CA, Dorsam RT, Mikelis CM, Masedunskas A, Amornphimoltham P, Nathan CA, Singh B, Weigert R, Molinolo AA, Gutkind JS (2011). Decreased lymphangiogenesis and lymph node metastasis by mTOR inhibition in head and neck cancer. Cancer Res.

[31] Hussein O, Tiedemann K, Murshed M, Komarova SV (2012). Rapamycin inhibits osteolysis and improves survival in a model of experimental bone metastases. Cancer Lett.

[32] Langley RR, Fidler IJ (2007). Tumor cell-organ microenvironment interactions in the pathogenesis of cancer metastasis. Endocr Rev.

[33] Gupta GP, Massagué J (2006). Cancer metastasis: building a framework. Cell.

